# Modelization of Blood-Borne Hypercoagulability in Myeloma: A Tissue-Factor-Bearing Microparticle-Driven Process

**DOI:** 10.1055/s-0039-1700885

**Published:** 2019-11-04

**Authors:** Loula Papageorgiou, Kutaiba Alhaj Hussen, Sandrine Thouroude, Elisabeth Mbemba, Héléne Cost, Laurent Garderet, Ismail Elalamy, Annette Larsen, Patrick Van Dreden, Meletios A. Dimopoulos, Mohamad Mohty, Grigoris T. Gerotziafas

**Affiliations:** 1 Research Group “Cancer, Haemostasis and Angiogenesis,” INSERM UMR_S 938, Centre de Recherche Saint-Antoine, Faculty of Medicine, Institut Universitaire de Cancérologie, Sorbonne Universities, Paris, France; 2 Service d'Hématologie Biologique Hôpital Tenon, Hôpitaux Universitaires de l'Est Parisien, Assistance Publique Hôpitaux de Paris, Paris, France; 3 INSERM U976, Université Paris-Diderot, École Pratique des Hautes Études/PSL Research University, Institut de recherche Saint-Louis, Hôpital Saint-Louis, Paris, France; 4 Clinical Research, Diagnostica Stago, Gennevilliers, France; 5 Research Group “Proliferation and Differentiation of Stem Cells” INSERM UMR_S 938, Centre de Recherche Saint-Antoine, Faculty of Medicine, Institut Universitaire de Cancérologie, Sorbonne Universities, Paris, France; 6 Department of Clinical Therapeutics, School of Medicine, National and Kapodistrian University of Athens, Athens, Greece; 7 Department of Hematology and Cell Therapy, Saint Antoine Hospital, Hôpitaux Universitaires de l'Est Parisien, Assistance Publique Hôpitaux de Paris, Sorbonne University, Paris, France

**Keywords:** multiple myeloma, thrombin generation, tissue factor, microparticles, hypercoagulability

## Abstract

**Introduction** Hypercoagulability is a common blood alteration in newly diagnosed chemotherapy naïve patients with multiple myeloma. The identification of the procoagulant potential of cancer cells, which is principally related to tissue factor (TF) expression, attracts particular interest. The mechanisms by which myeloma plasma cells (MPCs) activate blood coagulation have been poorly investigated.

**Aim** To identify the principal actors related with MPCs that boost thrombin generation (TG).

**Methods** TF and annexin V expression by MPCs and MPC-derived microparticles (MPC-dMPs) was analyzed by flow cytometry. TF activity (TFa) and TF gene expression were also determined. TG in the presence of MPCs or MPC-dMPs was assessed with the calibrated automated thrombogram assay (CAT) in normal human PPP and in plasma depleted of factor VII or XII. TG was also assessed in plasma spiked with MPCs and MPC-dMPs.

**Results** MPC-dMPs expressed approximately twofold higher levels of TF as compared with MPCs. The TFa expressed by MPC-dMPs was significantly higher compared with that expressed by MPCs. MPCs and MPC-dMPs enhanced TG of human plasma. TG was significantly higher with MPC-dMPs compared with MPCs.

**Conclusion** MPCs indirectly induce blood-borne hypercoagulability through the release of MPC-dMPs rich in TF. Since MPCs, expressing low TFa, represent a weak procoagulant stimulus, the hypercoagulability at the microenvironment could be the resultant of MPC-dMPs rich in TF.

## Introduction

Multiple myeloma is a plasma cell malignancy characterized by bone marrow infiltration leading to multiple lytic bone lesions, renal failure, anemia, and increased risk of venous thromboembolism. 1 Newly diagnosed, chemotherapy naïve patients with multiple myeloma present high levels of procoagulant phospholipids in plasma along with an increased concentration of biomarkers which indicate activation of blood coagulation and endothelial cells. 2

The identification of the procoagulant potential of cancer cells, principally mediated from tissue factor (TF), attracts particular interest since it is closely related with cancer aggressiveness, proangiogenic properties, resistance to anticancer treatment, and metastatic potential. 3 Enhanced fibrin formation as well as clots with low permeability and resistant to lysis have been observed in patients with multiple myeloma. 4 Myeloma plasma cells (MPCs) are potential initiators of the process leading to hypercoagulability. Fibrin together with activated platelets may either act as a shield of cancer cells against the access of anticancer drugs or alter the efficiency of the immunosurveillance system. 5 6 7 8 9

The crosstalk between cancer cells, plasma clotting mechanism, platelets, and endothelial cells enhances hypercoagulability. 10 Previous studies showed that mediators in this process vary according to the histological type of cancer cells. 11 12 Cancer cells from solid tumors induce thrombin generation by the expression of TF and the induction of factor XII (FXII) activation. 12 However, the intensity of the procoagulant potential varies according to the histological cancer cell type. 11 12 13 Cancer cells release procoagulant microparticles which have a major role in the amplification of their procoagulant potential and thrombin generation enhancement. 14 Recently, attention is being drawn on circulating extracellular vesicles released by cancer cells which are believed to mediate cell-to-cell communication. 15 From a conceptual point of view, MPCs would enhance hypercoagulability in their microenvironment. However, the interactions of MPCs with their microenvironment leading to blood coagulation activation have been poorly investigated. In the present study, we set up an experimental model that allows the identification of the procoagulant fingerprint of MPCs and MPC-derived microparticles (MPC-dMPs). In addition, this experimental model allows simulation of their impact on thrombin generation and elucidation of some aspects of the mechanisms by which MPCs induce hypercoagulability.

## Materials and Methods

### Human Plasma

Samples of fresh frozen normal platelet poor plasma (PPP; Ref 00539) and immunodepleted lyophilized plasma deficient of clotting factor VII (FVII) or FXII were purchased from Stago (Gennevilliers, France).

### MPCs and MPC-dMPs

#### Preparation of MPCs

**Fig. 1:**
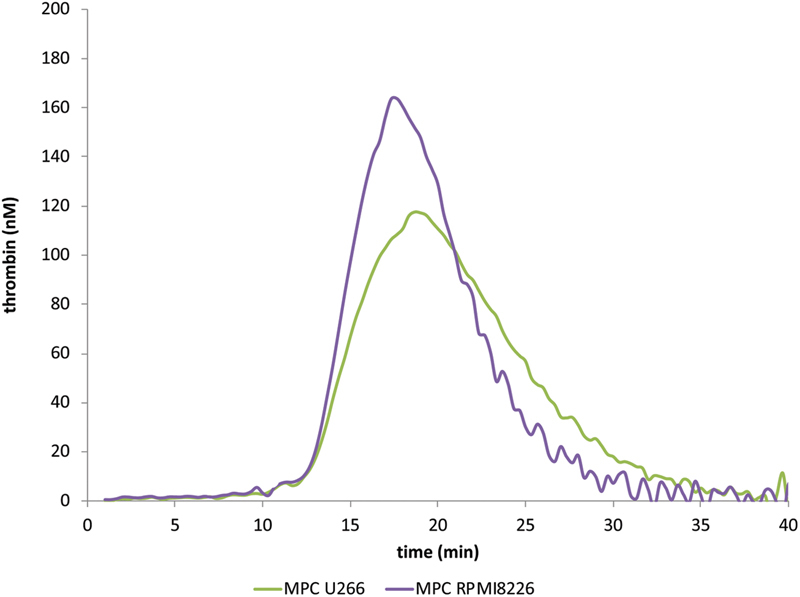
Representative thrombograms comparing thrombin generation triggered in the presence of MPCs (10 ^3^ /µL) RPMI 8226 or U266. Data are representative of five experiments. MPCs, myeloma plasma cells.

Human MPCs (RPMI 8226 and U266) obtained from American Type Culture Collection (ATCC; Rockville, Maryland, United States) were used. Human MPC lines grow in suspension. Cells were cultured in an RPMI 1640 medium (ATCC) supplemented with 10% (v/v) FBS and 1% (v/v) penicillin/streptomycin. Cell viability was assessed before each assay by trypan blue exclusion and cells with at least 90% viability were used. Experiments were conducted once a count of 1,000 cells/µL in the condition media was reached. At this point, 25 mL of MPCs suspension was centrifuged at 1,500 × g for 10 minutes at 25°C. Pellets of the MPCs were suspended at 1 mL of PPP yielding a MPC count of 25,000 cells/mL of PPP. Subsequently, serial dilutions of MPCs in PPP were performed and used in thrombin generation experiments. In preliminary experiments the procoagulant potential of the two MPC lines (RPMI 8226 and U266) was compared. Both cell lines showed similar procoagulant properties ( Fig. 1 ). For the sake of simplicity, we present herein the data from experiments performed on the RPMI 8226 cell line. The count of MPCs which produce a plateau effect on thrombin generation parameters was determined by constructing a “concentration–response” curve. The concentration of MPCs in PPP employed in most of the experiments (1,000 cells/mL) was situated at the plateau.

#### Preparation of MPC-dMPs

MPC-dMPs were isolated from the conditioned media of the MPC cultures by differential centrifugation. In brief, when the number of MPC in the conditioned media was 1,000 cells/μL and centrifugation for cell separation was performed as described above, 25 mL of supernatant was collected and was centrifuged at 1,500 g for 10 minutes at 25°C. The supernatant was collected and centrifuged at 20,000 g for 20 minutes at 4°C to pellet the MPC-dMPs. The pellets with MPC-dMPs were suspended in 1 mL of PPP. In preliminary experiments, “concentration–response” curves on thrombin generation assay were obtained to determine the count of MPC-dMPs which produced a plateau effect on thrombogram parameters. This procedure led to a sample preparation of a standardized MPC-dMP number, used for the experiments.

The procoagulant fingerprint of MPCs and MPC-dMPs was compared on the basis of the respective counts which were situated at the plateau of their effect on thrombin generation.

### Counting of MPC-dMPs

Microparticles were counted with the commercially available Zymuphen MP Activity assay purchased from Hyphen Biomed (Neuville sur Oise, France). The assay, performed according to manufacturer instructions, measured the procoagulant activity related to the concentration of phosphatidylserine (PS), and was performed using a microtiter plate sensitized with streptavidin and biotinylated annexin V, and then stabilized. Based on a functional assay, the concentration of microparticles was expressed at nM of PS. All experiments were performed with a standardized number of MPC-dMPs that corresponds to 26.2 nM of PS equivalent, derived from a MPC concentration of 1,000 cells/µL and situated at the plateau effect of the MPC-dMPs on thrombin generation. The employed concentration of MPC-dMPs (26 nM) was situated at the linear part of the plateau at the concentration–response curves obtained in the thrombin generation assay.

### Molecular Analysis of the Procoagulant Fingerprint of MPCs and MPC-dMPs

#### Specific TF activity

Cell suspensions (20 μL) of MPCs and MPC-dMPs were used at counts which produced a plateau effect on thrombin generation (1,000 cells/μL and 26.2 nM, respectively) were washed three times with phosphate-buffered saline (PBS), suspended in PBS and incubated at 4°C for 30 minutes. Subsequently, samples were centrifuged for 30 minutes at 1,000 g and supernatants were collected and kept frozen at −80°C until measurement of TF activity (TFa) as previously described. 16 TFa was also measured in normal plasma, the same used for thrombin generation experiments in which plasma cells or MPC-dMPs were suspended.

#### Flow Cytometry

Cell suspensions (20 μL) of MPCs and MPC-dMPs were used at counts which produced a plateau effect on thrombin generation (1,000 cells/μL and 26,2 nM, respectively). MPCs were suspended in PBS, and centrifuged at 1,500 g for 10 minutes at 25°C. For TF assessment, cells resuspended in PBS were incubated with 10 μL of a control mouse immunoglobulin IgG1 FITC (BD Pharmingen, 555748) and with IgG1 PE (Beckman Coulter, A07796, France) or with a mouse monoclonal antibody against human TF FITC (Sekisui Diagnostics, United States, 4507CG) and with an anti- CD138 PE (Beckman Coulter, Ref A54190, France), for 30 minutes at room temperature in the dark. After incubation, cells were washed once with PBS and then suspended in PBS for TF assessment. Sample data were acquired, collected, and analyzed using an FC 500 flow cytometer (Beckman Coulter, Villepinte, France) with CXP Acquisition (Beckman Coulter, Miami, Florida, United States) and Flow JO Analysis software (TreeStar Inc). For the standardization of microparticles by flow cytometry, an initial microparticle-size gate was set with the help of Megamix (Biocytex, Marseille, France, 7801), a mixture of microbeads of three different sizes (3.0, 0.5, and 0.9 μm) which was developed to confirm microparticles' size. To separate true events from background noise and unspecific binding of antibodies to debris, we defined microparticles as particles that were between 0.5 and 0.9 μm in diameter and we explored whether they presented positive staining for TF and/or annexin V. For annexin V staining, the microparticle suspension was incubated for 30 minutes with 10 µL PE–annexin V (kit, BD Pharmingen, 559763). Annexin V–PE with PBS without calcium was used as control. TF staining was done as in cells. Analyses were performed on a flow cytometer (Navios) using a Megamix bead-calibrated protocol (BioCytex).

#### Polymerase Chain Reaction

Total RNA was extracted from MPCs and reversely transcribed according to a standardized procedure; briefly, 1 μg cDNA was amplified using the Go TAq 180 qPCRMaster mix (Promega) and the Mx3000P qPCR system according to the manufacturer's protocol. Annealing temperature was 60°C. Quantitative reverse transcriptase PCR (RT-PCR) was used to measure the relative mRNA levels using TaqMan assays for TF (Sigma Aldrich). The following primers were used: TF L,5′ AGG GTC TTC ATG CTC CGA AA 3′, TF U,5′ TGT AGA AAG GCA GGA CTG GG 3′. The reaction was inhibited after 40 cycles. *Ct* values were normalized against the endogenous control B-actin (from Sigma Aldrich). Relative mRNA expression was calculated using the comparative threshold method (2 ^−ΔΔ *Ct*^ ).

### Calibrated Automated Thrombogram Assay

In each well of a 96-well microplate, a volume of 80 µL PPP was spiked with 20 µL of PPP containing MPCs or MPC-dMP suspension prepared and counted as described in the previous paragraphs. Counts of MPCs and MPC-dMPs in the PPP at the wells of the microplate are fivefold lower than the initial counts. Thrombin generation was initiated by adding a 20 μL triggering solution containing CaCl _2_ (16.7 mM final concentration) and a fluorogenic substrate (Z-Gly-Gly-Arg-AMC, 417 μM final concentration). Thrombin generation was assessed with the Calibrated Automated Thrombogram according to the manufacturer instruction. Among all thrombogram parameters, we analyzed lag time, peak of thrombin, and the mean rate index (MRI). The last one reflects the rate of the propagation phase of thrombin generation [calculated by the formula: MRI = peak/(ttPeak – lag time)]. The acquisition time of thrombogram parameters was defined at 40 minutes.

In separate experiments, thrombin generation in 80 μL of normal PPP (without any exogenous addition of MPCs or MPC-dMPs) was performed using 20 μL of MP-reagent (4 μM procoagulant phospholipids), or PPP-reagent Low (1 pM TF and 4 μM procoagulant phospholipids), or PPP-reagent (5 pM TF and 4 μM procoagulant phospholipids). Thrombin generation was performed according to manufacturer instructions as described above.

All reagents were from Stago (Gennevilliers, France). The control consisted of a mixture of 80 μL PPP and 20 μL NaCl. All counts of MPCs and MPC-dMPs mentioned in thrombin generation experiments refer to the mother suspensions.

### Thrombin Generation in the Presence of an Anti-TF Antibody

In separate experiments, 50 μL of a working suspension of MPCs (500 cells/µL) in normal PPP was mixed with an equal volume of PPP containing an anti-TF mouse monoclonal antibody 4509 (American Diagnostica, Neuville-sur-Oise, France). Mouse IgG1 isotype (100 μg/mL) or NaCl was used in control experiments. Cells were incubated with the anti-TF antibody or the isotype IgG1 for 15 minutes at 37°C. The experimental conditions were defined after conducting preliminary experiments on thrombin generation, with variable concentrations of the cells and the anti-TF monoclonal antibody. Cells were used at the lower active concentration in plasma and the antibody was used at a concentration of 25 μg/mL. At this concentration the anti-TF antibody completely inhibited the effect of the PPP-reagent (5 pM TF and 4 μM procoagulant phospholipids) on thrombin generation.

### Statistical Analysis

All experiments were performed for at least five times. Results are shown as mean ± standard deviation. Data were normally distributed, and a paired sample *t* -test was applied to compare parameter differences in all methods. The level of statistical significance was set at 0.05. Two-sided values of *p* < 0.05 were considered as statistically significant. The SPSS statistical package (version 21.0 IBM) was used for statistical analysis.

## Results

### Tissue Factor and Procoagulant Phospholipid Expression by MPCs and MPC-dMPs

**Fig. 2:**
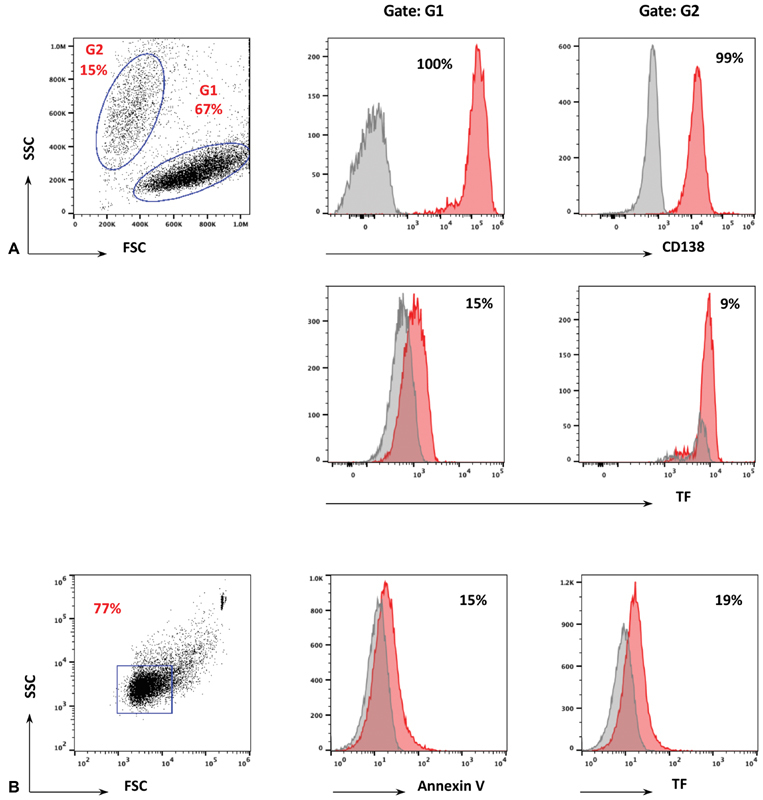
Tissue factor (TF) expression in myeloma plasma cells (MPCs). RPMI 8226 cell lines of MPCs were cultured in RPMI medium, 10% FBS, and 1% penicillin–streptomycin, and flow cytometry analysis for TF expression was performed as described in Materials and Methods in MPCs (frame A) or in MPC-dMPs (frame B) using the following antibodies: PE CD138, FITC TF, and PE annexin V. Data are representative of three experiments. MPC-dMPs, myeloma plasma cell-derived microparticles.

**Fig. 3:**
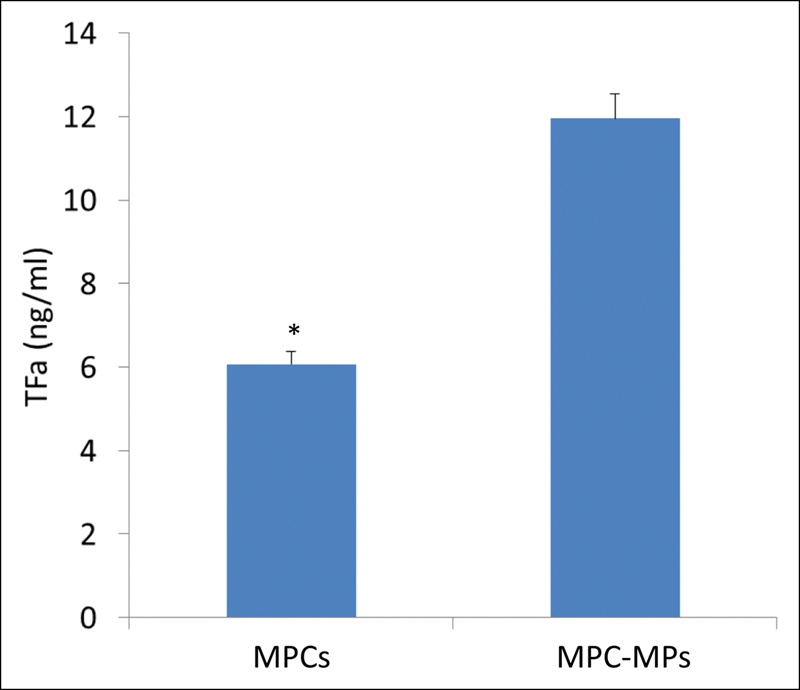
TFa expression by MPC-dMPs was significantly higher compared with that by myeloma plasma cells (* *p* < 0.05). Values are means ± SD of three experiments. MPC-dMPs, myeloma plasma cell-derived microparticles; SD, standard deviation; TFa, tissue factor activity.

Flow cytometry analysis showed that MPC-dMPs expressed approximately twofold higher levels of TF as compared with MPCs ( *p* < 0.05) ( Fig. 2 , frames A and B). MPC-dMPs were positive for the expression of procoagulant phospholipids, whereas this was not the case for the MPCs ( Fig. 2 , frame B). MPCs and MPC-dMPs possessed specific TFa. The TFa expressed by MPC-dMPs (26.2 nM) was significantly higher as compared with that expressed by MPCs at the count of 1,000 cells/μL (11.95 ± 1.9 and 6.07 ± 0.4 ng/mL, respectively; *p* < 0.05; Fig. 3 ). The PCR analysis showed an expression of TF gene by MPCs (relative expression of 0.178).

## Procoagulant Potential of Plasma Cells and MPC-dMPs

### Impact of Plasma Cells on Thrombin Generation

MPCs induced a significant decrease of the lag time and a significant increase of the MRI and peak as compared with the control experiment (PPP without any cell addition; Table 1 ). MPCs at counts equal or higher than 250 cells/µL had a significant impact in lag-time reduction. A plateau effect was observed at MPCs counts equal or higher than 500 cells/µL (data not shown).

**Table 1:** Effect of MPCs (1,000 cells/μL) or MPC-dMPs on thrombin generation in normal PPP and in PPP depleted of FVII or FXII

Thrombogram parameters	Normal PPP							FVII deficient			FXII deficient		
	4 µM procoagulant phospholipids			No procoagulant phospholipids				No procoagulant phospholipids			No procoagulant phospholipids		
	5 pM TF	1 pM TF	No TF	1 pM TF	No TF	MPCs	MPC-dMPs	No TF	MPCs	MPC-dMPs	No TF	MPCs	MPC-dMPs
Lag time (min)	3 ± 1.2	4.7 ± 0.9	13.8 ± 3.1	9.2 ± 0.6	17 ± 0.6	8 ± 1.7 a b	6 ± 1.1 a b	> 44	> 44	> 44	> 44	17 ± 2.1 c	13 ± 1.8 c
Peak (nM)	186 ± 5.6	89.1 ± 12.1	81.4 ± 2.1	46.9 ± 11.5	73 ± 5.6	120 ± 30 a b	200 ± 22.4 a	0	0	0	0	28 ± 1.7 c	84 ± 3.2 c
MRI (nM/min)	53 ± 1.6	52.4 ± 45.7	47.6 ± 3.2	5.8 ± 3.1	15 ± 2.4	34 ± 15 a b	72 ± 20.4 a	0	0	0	0	28 ± 2.6 c	19 ± 0.6 c

Abbreviations: FVII, factor VII; FXII, factor XII; MPCs, myeloma plasma cells; MPC-dMPs, myeloma plasma cell-derived microparticles; MRI, mean rate index; PPP, platelet poor plasma.

Note: PPP-reagent, 5 pM of TF, and 4 μM procoagulant phospholipids. Comparison with thrombin generation triggered in the presence of standard concentrations of TF and procoagulant phospholipids. Values are the mean ± SD of five experiments.

a *p* < 0.05 versus PPP + CaCl _2_ .

b *p* < 0.05 versus PPP + PPP-reagent.

c *p* < 0.05 versus def-FXII + CaCl _2_ .

In order to explore which pathway of blood coagulation is activated by MPCs, experiments were done in plasma depleted of FVII or FXII. In the control experiment (PPP + CaCl _2_ ), no detectable thrombin generation was observed in the absence of any of the two clotting factors. The MPCs (1,000 cells/μL) suspended in FVII-deficient plasma did not induce any detectable thrombin generation. In contrast, MPCs suspended in FXII-deficient plasma induced thrombin generation ( Table 1 ).

### Impact of MPC-dMPs on Thrombin Generation

MPC-dMPs induced a significant decrease of the lag time and a significant increase of the MRI and peak, as compared with the control experiment (PPP without any MPCs or MPC-dMP addition). MPC-dMPs at counts equal or higher than 20 nM had a plateau effect on thrombin generation.

In order to explore which pathway of blood coagulation is activated by MPC-dMPs (26.2 nM), experiments were done in plasma depleted of FVII or FXII. MPC-dMPs suspended in FVII-deficient plasma did not induce any detectable thrombin generation. Suspension of MPC-dMPs in FXII-deficient plasma led to thrombin generation. Data are summarized in Table 1 .

### Comparison of the Procoagulant Activities of MPCs and MPC-dMPs to Standard Concentration of TF and Phospholipids

**Fig. 4:**
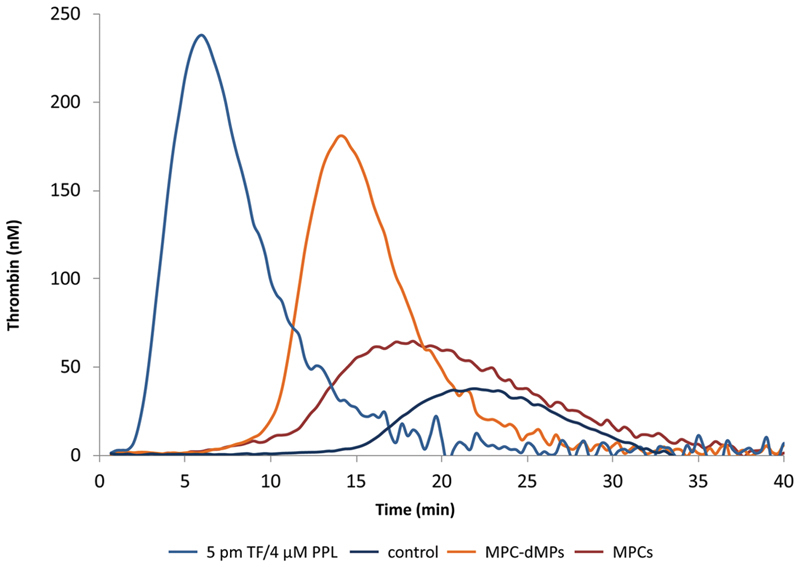
Comparison of thrombin generation triggered in the presence of MPCs or MPC-dMPs in normal human PPP versus that triggered by 5 pM TF and 4 µM procoagulant phospholipids. Thrombin generation in recalcified PPP without any exogenous addition of procoagulant trigger is also depicted. Representative thrombograms of one out of five experiments. MPCs, myeloma plasma cells; PPP, platelet poor plasma.

In the presence of MPCs (1,000 cells/µL), the lag time was significantly longer, and the peak and MRI were significantly lower as compared with those observed in the presence of standard preparations of TF (5 or 1 pM) and procoagulant phospholipids (4 μM, respectively). MPC-dMPs (26.2 nM) induced a higher peak and MRI as compared with the standard preparations of TF and procoagulant phospholipids. Representative thrombograms are shown in Fig. 4 .

Both MPCs and MPC-dMPs significantly enhanced thrombin generation as compared either with that induced by 1 pM of TF without any additional phospholipids or with that induced by procoagulant phospholipids (4 µM) in the absence of TF ( Table 1 ).

### Impact of Anti-TF Monoclonal Antibodies on Thrombin Generation Induced by MPCs and MPC-dMPs

The addition of anti-TF monoclonal antibodies to PPP with MPCs and MPC-dMPs significantly prolonged the lag time and ttPeak and decreased the MRI and the peak of thrombin generation compared with control experiments without anti-TF antibodies. Interestingly, the relative inhibition of thrombin generation was higher for MPCs compared with MPC-dMPs ( Table 2 ).

**Table 2:** Impact of TF inhibition by a specific anti-TF antibody on thrombin generation in normal PPP induced by MPC or MPC-dMPs

	NaCL + anti-TF	NaCL + IgG	MPCs + IgG	MPC-dMPs + IgG	MPCs + anti-TF	MPC-dMPs + anti-TF
Lag time (min)	> 44	> 44	7.9 ± 1.5	6.5 ± 1.3	> 44 a	20 ± 3.2 a
Peak (nM)	0	0	118 ± 33	186 ± 25	0 a	40 ± 12 a
MRI (nM/min)	0	0	32 ± 13	68.2 ± 19	0 a	12 ± 1 a

Abbreviations: IgG, Immunoglobulin G; MPC, myeloma plasma cell; MPC-dMPs, myeloma plasma cell-derived microparticles; MRI, mean rate index; PPP, platelet poor plasma; TF, tissue factor.

Note: Values are the mean ± SD of three experiments.

a *p* < 0.001 versus the control experiments (MPC + IgG and MPC-dMPs + IgG, respectively).

## Discussion

It is well established that cancer cells express TF, the major trigger of blood coagulation. 3 The levels of TF expressed by cancer cells enhance thrombin generation principally via the TF pathway, whereas their procoagulant potency varies according to their histological type. 11 12 13 The present study investigates the interactions of MPCs with the blood coagulation process and particularly with the thrombin generation process. Two issues related to the procoagulant fingerprint of MPCs were investigated: (1) the intensity of the procoagulant potential expressed by MPCs and MPC-dMPs and (2) the evaluation of the relative roles of the TF pathway and the intrinsic pathway of blood coagulation activation initiated by MPCs and MPC-dMPs.

In MPCs, the expression of TF gene, the presence of TF protein on cell membrane, and the procoagulant activity of TF are detectable. Comparison with data previously published by our group using the same experimental model 11 12 13 confirms that MPCs express significantly lower TF as compared with cancer cells from solid tumors such as breast cancer cells MCF7 and pancreatic cancer cells BXPC3.

MPCs release microparticles into their microenvironment which show marked procoagulant activity. Indeed, the MPC-dMPs express approximately twofold higher levels of TF as compared to their origin cells. Accordingly, the activity of TF expressed by MPC-dMPs is about twice higher as compared with that of MPCs. In addition, MPC-dMPs show significant expression of procoagulant phospholipids.

MPCs accelerated the initiation phase (lag-time decrease) and amplified the propagation phase (MRI increase) of thrombin generation. This resulted in a higher thrombin peak compared with the basic thrombin generation potential of normal plasma. However, the potency of MPCs to generate thrombin was significantly lower as compared with that induced by concentrations of 5 pM TF and 4 µM of procoagulant phospholipids, which are considered to be the concentration of TF and procoagulant phospholipids estimated to be exposed during normal hemostasis following vascular injury. 17 Thus, the procoagulant potential of MPCs appears to be lower than that attained from the normal function of blood coagulation system. In contrast, MPC-dMPs showed a more potent effect on thrombin generation as compared with their cells of origin. Assessment of thrombin generation in FVII- or FXII-deficient plasma allowed the evaluation of which clotting pathway is activated by MPCs and MPC-dMPs. The complete abrogation of thrombin generation in plasma depleted of FVII reveals that the contact system and FXII activation have a very limited role—if any—on thrombin generation induced by MPCs. The persistence of thrombin generation in plasma depleted in FXII further demonstrates the importance of the TF/FVIIa pathway activation by MPCs. The experiments with the specific anti-TF antibody showed that both MPCs and MPC-dMPs induced thrombin generation in a TF-dependent manner. However, taking into consideration that the antibody used selectively inhibits the procoagulant activity of TF, we could not exclude that some of the procoagulant properties of the MPCs and MPC-dMP are due to the signalization function of TF.

Based on data presented herein, we conclude that MPCs and MPC-dMPs activate blood coagulation principally via the TF pathway activation. The contact system activation has a secondary role in this process.

These data lead to the concept that myeloma-related hypercoagulability is the resultant of the MPCs procoagulant properties combined with the procoagulant elements of the microenvironment. Following this concept, we assume that MPC-dMPs released by MPCs into the microenvironment enhance the weak procoagulant activity of MPCs. This weak procoagulant stimulus of MPCs and the stronger one expressed by the MPC-dMPs have an impact on the formation of a local fibrin network and could have a protector effect from both the immunosurveillance system and the access of antimyeloma treatment. All the above are issues that merit further investigation.

Extrapolation of the findings of this study in patients should be cautious. The present study provides evidence for the modelization of the procoagulant interactions between the MPCs and their plasma microenvironment and shows that the MPC-dMP is the principal vector of hypercoagulability.

To the best of our knowledge, the present study reports for the first time that MPCs function as a weak stimulus of thrombin generation but amplify the procoagulant stimulus by releasing MPC-dMPs. The MPC-dMPs, which express about a twofold higher amount of TF as compared with MPCs as well as procoagulant phospholipids, induce a TF-dependent activation of blood coagulation and are the principal vectors of the procoagulant stimulus in the plasma microenvironment. Due to their critical role in hypercoagulability, procoagulant MPC-dMPs could be a potential target for the evaluation of both the biological activity of MPCs and the assessment of the thrombogenic potential.
